# Editorial: Proteomics as a Tool for Biomarker and Drug Target Discovery: Improving the Diagnosis and Treatment of Neurodegenerative Diseases

**DOI:** 10.3389/fnagi.2020.00232

**Published:** 2020-11-26

**Authors:** Olivia Belbin, Sylvain Lehmann, Eduard Sabidó, Christophe Hirtz

**Affiliations:** ^1^Biomedical Research Institute Sant Pau (IIB Sant Pau), Barcelona, Spain; ^2^Centro de Investigación Biomédica en Red sobre Enfermedades Neurodegenerativas (CIBERNED), Madrid, Spain; ^3^IRMB, University of Montpellier, INSERM, CHU Montpellier, (LBPC-PPC), Montpellier, France; ^4^Proteomics Unit, Center for Genomics Regulation, Barcelona Institute of Science and Technology (BIST), Barcelona, Spain; ^5^Proteomics Unit, Universitat Pompeu Fabra, Barcelona, Spain

**Keywords:** proteomics, biomarker, drug targets, neurodegenerative diseases, Alzheimer's disease, Parkinson's disease, amyotrophic lateral sclerosis, frontotemporal dementia

As the number of people surviving beyond their 80s is growing dramatically, the prevalence of age-related neurodegenerative diseases is expected to almost double over the next 20 years, representing one of the major health challenges facing modern society. A common barrier to tackling these devastating diseases is that there are currently no drugs that slow down or stop their progression, and there is an urgent need for specific biomarkers that inform of disease evolution prior to symptom onset, when drugs are more likely to be effective.

Recent technological advances have led to a gradual adoption of proteomic strategies to identify proteoforms involved in early disease pathways for use as drug targets and/or diagnostic markers. This collection of original research and review articles showcases how novel and state-of-the-art proteomics approaches such as Parallel Reaction Monitoring (PRM), Multiple Reaction Monitoring (MRM), Isobaric Tags for Relative and Absolute Quantitation (iTRAQ), Terminal Amine Isotopic Labeling of Substrates (TAILS), to name just a few, can be effective in identifying novel biomarkers and drug targets for the most prevalent neurodegenerative diseases, namely Alzheimer's disease (AD), Parkinson's disease (PD), amyotrophic lateral sclerosis (ALS), and frontotemporal dementia (FTD).

AD represents the most prevalent neurodegenerative dementia (World Health Organization, 2017) and is characterized by the deposition of aggregated β-amyloid peptide (Aβ) and hyperphosphorylated tau protein. While Aβ and tau, measured in CSF, make excellent surrogate markers of underlying brain pathology in AD, standard immunoassays do not reflect changes in tau phosphorylation rate, a phenomenon that seeds intraneuronal tau aggregation (Alonso et al., 2001). Here, Barthélemy et al. report over 29 distinct phosphorylation sites in brain-derived tau and 12 sites in CSF-derived tau that showed variable abundance between AD patients and non-AD controls. This study is a strong example of how multiplexed quantification can be used to profile post-translational modifications that are otherwise undetected by standard immunoassays. These pathological species may prove valuable for improving AD diagnosis and tracking therapeutic responses in AD patients.

Reduced neuronal connectivity occurs early in AD (Selkoe, 2002) and can be detected in living patients by neuroimaging methods that quantify glucose metabolism. However, reliance on radioactive tracers and other limitations of this technique preclude its use in all patients. Consequently, there is a need for an alternative marker of metabolic dysfunction that can be detected in patient biofluids. Here, Bergau et al. employed MRM to monitor 122 metabolites in CSF from AD patients and controls. They identified 4 glucose metabolites that were significantly reduced in AD and that correlated with amyloid pathology. This direct targeting of metabolic pathways resulted in the identification of a novel biomarker with improved diagnostic sensitivity (95.5%) and specificity (93.7%) for AD.

Smell impairment is another early event in AD (Attems et al., 2014; Daulatzai, 2015). However, little is known about the initial molecular disturbances in the olfactory bulb. Here, Lachen-Montes et al. combined iTRAQ labeling and transcriptomic analyses to study metabolomic changes in the olfactory bulb from the Tg2576 AD mouse model in a unique window of time that would not be possible using human subjects. They show that overproduction of human mutated amyloid precursor protein altered olfactory signaling in these mice even prior to the appearance of amyloid pathology. Survival kinome profiling revealed concomitant changes in the activation dynamics of two olfactory pathways with memory deficits and AD pathology. Both pathways were altered in postmortem olfactory bulb tissue from AD patients. By targeting a region that is affected early in AD, the authors identified two dysfunctional pathways, whose monitoring in living patients may improve early diagnosis and lead to novel therapeutic targets for AD.

PD is the second most frequent neurodegenerative disease worldwide. As there are currently no early diagnostic biomarkers for PD, improved understanding of the earliest mechanisms is needed. PD is characterized by the specific loss of nigral dopaminergic neurons (Obeso et al., 2017), which are particularly vulnerable to mitochondrial dysfunction (Strauss et al., 2005; Shanbhag et al., 2012). Here, Lualdi et al. developed a strategy termed “mitochondrial dimethylation-TAILS” to analyze the mitochondrial N-terminome in a neuroblastoma cell line adapted to model dopamine dyshomeostasis. They achieved 40% coverage of the mitochondrial proteome, an improvement over classical shotgun methods. They reported 11 mitochondrial N-terminal peptides whose levels were affected by dopamine treatment, and identified the protease Neprilysin as a promising surrogate marker of early mitochondrial dysfunction in PD.

FTD and ALS share common clinical, pathological, and genetic features (Ferrari et al., 2011). A major challenge in diagnosing these diseases is the variable correspondence between the clinical syndrome and the underlying neuropathological disorders (Josephs et al., 2011; Irwin et al., 2015). FTD diagnosis is further confounded by a potential overlap in the clinical phenotype with AD and psychiatric disorders. Here Hedl et al., present a comprehensive review of published studies that have taken a proteomics approach to biomarker and drug target discovery in the FTD-ALS spectrum. They discuss how peptides from stable isotope-labeled (SILAC) human embryonic kidney 293 cells have been used as internal standards to allow quantification of low-abundant proteins across control and FTD brain proteomes (Seyfried et al., 2012) and how Tandem Mass Tagging (TMT) has identified proteome-wide nucleocytoplasmic changes and altered RNA stability in cell-based models of ALS (Kim et al., 2017; Tank et al., 2018). They also highlight how unbiased approaches such as label-free quantitative proteomics or Sequential Windowed Acquisition of all THeoretical fragment ion Mass Spectra (SWATH-MS) have been employed to identify disease-related changes in specific pathways (Iridoy et al., 2018; Umoh et al., 2018; Xu et al., 2018) and to classify distinct subtypes of ALS and FTD (Laferriere et al., 2019). Overall, these studies offer great hope for the development of biomarkers and therapeutics for FTD and ALS.

The aim of this collection is to stimulate conversation and advance research toward the prevention of these devastating neurodegenerative diseases. The collection highlights the wide range of technological strategies and source material available, each with its own advantages. These studies not only advance our understanding of the biological basis of these diseases, but also provide several promising avenues for that could improve diagnosis and disease management of the most common neurodegenerative diseases.

## Author Contributions

OB, SL, ES, and CH edited this Research Topic and all contributed to the drafting of this editorial.

## Conflict of Interest

The authors declare that the research was conducted in the absence of any commercial or financial relationships that could be construed as a potential conflict of interest.
